# Urban Churches Show an Increase in Attendance, Donations, and Finances During the COVID-19 Pandemic in the USA: Evidence from the United Methodist Church

**DOI:** 10.1007/s10943-024-02046-z

**Published:** 2024-05-06

**Authors:** Anum G. Niazi, Nayab Ahmed, Shandana Kifayat, Shanlina Kifayat, Mohammad Asad Niazi, Muhammad Salar Khan

**Affiliations:** 1 Johns Hopkins Bloomberg School of Public Health, Baltimore, MD USA; 2 Clear Cognition Solutions, London, UK; 3 Khyber Teaching Hospital, Khyber Medical University, Peshawar, Pakistan; 4 Lady Reading Hospital, Peshawar, Pakistan; 5 CUNY Brooklyn College, Brooklyn, NY USA; 6 Schar School of Policy and Government, George Mason University, Fairfax, VA USA

**Keywords:** COVID-19, Religion, United Methodist Church, Congregations, Donations, Finances, USA

## Abstract

The COVID-19 pandemic has had a significant impact on religion and its practice. This paper aims to examine how the pandemic affects religious activities, donations, and finances over time and across regions within the United Methodist Church (UMC) in the USA. To address this question, we analyze survey data collected during the pandemic from 2963 churches in the USA by United Methodist Communications. Our analysis utilizes several quantitative techniques, including Z-tests, one-way analysis of variance (ANOVA), and multinomial logistic regressions. The results indicate a decrease in church attendance over time, with a more pronounced effect observed in non-urban areas (suburban, small town, and rural). Similarly, while church donations and finances mitigate over time across churches, churches in urban areas experience a quicker rebound compared to those in non-urban areas. Lastly, we find that church attendance and donations positively affect finances. These findings hold important implications for churches in various regions, offering insights to develop strategies for navigating the challenges posed by the COVID-19 pandemic.

## Introduction

COVID-19 has affected both individuals and organizations in multiple ways. While researchers have examined the effects of the pandemic on health and the economy, the impacts on attendance and economic activities of religious organizations have been explored less. Religion is an imperative component of the lives of many across the world. Approximately 80% of Americans classify themselves as following a religion.^1^ Similarly, about 84% of the world’s population believes in a religion.^2^ Since a large proportion of the world’s population follows a religion, it is important to examine the effects of the pandemic on religious activities and finances to understand how people are adapting. Therefore, in this paper, we examine how the COVID-19 pandemic affects the activities such as attendance, donations, and finances of the United Methodist Church (UMC) in the USA.

Hypothetically speaking, social and logistical disruptions caused by a pandemic could alter the practice of religion. For instance, people’s perception of religion as a moderating force offering solace and communal space may increase religious rituals and finances. Alternatively, religious activities and finances may decrease if people felt disconnected from religion. The disruption of attendance in houses of worship during the pandemic can further decline religious activities and finances. Geography may also play a role in the practice of religion. It will be interesting to explore these trends for urban vis-à-vis rural churches.

Historically, a positive correlation is apparent between calamities and religious beliefs and practices, as observed in the 1918 flu in South Africa, the outbreak of smallpox in India, and the terrorist attacks in 2001 in the USA (Ai et al., 2005; DeFranza et al., 2020; Osheim, 2008; Poulin et al., 2009; Torabi & Seo, 2004).

However, compared to previous calamities, the impact of COVID-19 on health and the economy is far greater. The extent of the pandemic’s effect on religion and its practice remains largely unknown. In addition to examining the effect of the pandemic on individual religious activities, it is important to consider the implications of the pandemic on the economics of religious organizations, which play a pivotal role in providing employment and contribute to society by offering mental consolation, facilitating medical assistance (such as disbursing medicines and equipment like masks), and serving as social centers (providing food and shelter to the poor and needy). The multifaceted importance of religious organizations has further increased during the pandemic.

Despite the immense significance of religious organizations, little is known about their financial vulnerability during the ongoing pandemic. Recognizing the importance of religion as a source of solace for many individuals and the organizational significance of religious institutions, we aim to investigate the effects of the pandemic on three crucial aspects of the UMC in the USA: 1) UMC attendance, 2) church donations, and 3) the church’s financial condition. The investigation will encompass different time periods and regions.

Our study broadly contributes to literature on the practice of religion in religious organizations and COVID-19 in at least three important ways. First, little is known about how the nature and extent of challenges faced by religious organizations shaped up as the pandemic prolonged. Therefore, we investigate the temporal effects of the pandemic on three aspects of the church (attendance, donations, and finances) by examining them at three distinct time points during the pandemic.

Second, as mentioned earlier, historically, there has been a positive correlation between calamities and religious practices. However, the scale and nature of the COVID-19 pandemic is unique. Thus, it will be interesting to see whether the historically observed positive relationship between pandemic and religious practice persists in the modern age of technology and religious moderation, as challenged by precautions such as social distancing and lockdowns restricting social events.

Third, in contrast to most existing studies on pandemics and religion (Frei-Landau, 2020; Kowalczyk et al., 2020; Williams et al., 2021), we use unique and rich data collected by the United Methodist Communications (UMCom) within the UMC in the USA. As the data elicit information directly from pastors and church laity, we identify the precise effects of the pandemic on our three outcome variables (i.e., church attendance, donations, and finances). The estimated effects are relatively reliable because the information acquired from pastors by their internal organization is less likely to be affected by possible biases caused by factors such as the surveyor effect, image concerns, and peer pressure, observed in self-reported individual-level religiosity data (Sablosky, 2014; Umer, 2020). Additionally, reported religiosity can differ from actual religiosity, but the information provided by pastors addresses these issues as they provide insights into actual religious activities.

The rest of the paper is organized as follows: Section "Related Literature" discusses related literature, while Sect. "Data explanation" describes the data source and provides an overview of the variables used in the analysis. Section "Methods" reports the statistical methods employed. In Sect. "Statistical analysis and results", we present and discuss the statistical analyses and results for the three outcome variables (church attendance, church donations, and church finances), while Sect. "Discussion and implications" further elaborates on the results and their implications. Section "Limitations and suggestions for further research" highlights limitations and offers suggestions for further research. Finally, Sect. "Conclusions" concludes the paper.

## Related Literature

Previous research has documented how social changes brought by COVID-19 impacted religion and vice versa (Baker et al., 2020; Brien, 2020; Domaradzki, 2022; Molteni et al., 2021; Village & Francis, 2021). For instance, one study utilized shelter-in-place directives as an intervention to analyze adherence to COVID-19 protocols in relation to religiosity over a period of 30 days (DeFranza et al., 2020). The results indicated that religiosity had no influence on individuals’ movements when shelter-in-place directives were not in effect. However, when the directives were implemented, higher levels of religiosity were associated with reduced adherence to the directives.

Some studies also discuss how demographics, particularly age and gender, influenced the practice of religion (James et al., 2020; Kowalczyk et al., 2020). For example, a survey study conducted in Poland, which involved 324 participants, revealed a predominance of young individuals, both men and women having an interest in religion during the pandemic (Kowalczyk et al., 2020). Among the young age-group (21–35 years), faith was deemed significant, with frequent prayer practice. Authors of this research note that in Poland, the discourse on the increasing crisis of faith among the younger generation and their detachment from church traditions makes the surveyed group a captivating case for further investigation, given their lack of prior exposure to such a societal crisis. This research also provides evidence that women tend to express a greater inclination toward strengthening their faith or spirituality in the face of the COVID-19 threat.

Additionally, many studies discuss the role of religion in health promotion during the pandemic (Barmania & Reiss, 2021; Hashmi et al., 2020; Koenig, 2020; Modell & Kardia, 2020; Ribeiro et al., 2020). One such study that focused on the involvement of religion in health promotion presented with a dual perspective (Modell & Kardia, 2020). On the one hand, religion served as a vital source of hope, providing emotional support and sustaining lives during the COVID-19 crisis. On the other hand, religious institutions offered practical services that contribute to overall health and welfare. Although many individuals identify as more spiritual than religious, it is the organized nature of religious institutions that is playing a crucial role in addressing widespread financial and food shortages, which assists in overall welfare and health of the populations.

Further speculation into the role religion played during the COVID-19 pandemic was highlighted by a survey study conducted online with 1,173 Australian churchgoing Catholics, using the *Spiritual Well Being Scale*, found no notable difference in the scores for “Existential and Religious Wellbeing” between two groups: those who were still experiencing church closures and those who were able to attend church again (Kretzler et al., 2023). Among those who were still facing church closures, a standard multiple regression analysis revealed a significant and positive correlation between age and engagement in virtual worship, as well as higher scores in both Existential and Religious Wellbeing. However, for those who were able to attend church again, the frequency of Mass attendance before and after closures and participation in virtual and in-person worship during closures were all predictors of higher scores in Existential and Religious Wellbeing. These findings support the importance of providing access to both virtual and in-person worship during church closures, as they have positive effects on existential and religious well-being among churchgoing Catholics.

As is well-known, churches rely on large congregations; however, studies suggest that these sites are likely to be key for COVID-19 transmission (Hamner, 2020; James et al., 2020; Vermeer & Kregting, 2020; Yong et al., 2020). Following the peak of the pandemic in early Spring 2020, churches were closed to prevent in-person transmission (Perry & Grubbs, 2022). With most church activities shifting online and a surge in asynchronous communication, the impact of peers, group cohesion, and feedback through physical interaction has been tremendously altered or reduced (Baker et al., 2020). This, along with “online fatigue,” may have further influenced online church attendance levels during the pandemic (Baker et al., 2020).

Similarly, finances are a constant concern for churches. Like church attendance, COVID-19 has posed challenges to the church’s ability to raise finances and donations (Mulder & Martí, 2020). In fact, finances and donations are linked to attendance (Maftei, 2020). Churches count on Sunday worship and events like Easter and Christmas, which are big donation days for churches because of the attendance spikes they usually experience (Maftei, 2020). With Easter falling during the peak of the pandemic in the USA, it may have significantly affected all churches, particularly those in suburbs, small towns, and rural areas that did not have robust online platforms for raising donations. Many media articles discuss how COVID-19 has hit church finances and donations in the USA.^3^

These studies lay the groundwork and enhance our comprehension of the role played by religion during the COVID-19 pandemic, as well as how the pandemic moderated the practice of religion. However, it is important to note that these studies mostly cover Europe and Australia, and their findings are based on smaller samples. While there are opinions and speculations regarding church activity levels and finances in the USA (i.e., Estrin, 2022), there is a scarcity of systematic empirical studies that encompass these aspects specifically during the pandemic, which will be the focus of our work. Ideally, we would like to include many religious groups, but data availability limits us to conducting this analysis specifically on the United Methodist Church in the USA.

## Data Explanation

Our data covers the United Methodist Church (UMC) in the USA. Before delving into data details, it is important to mention why we chose USA for this analysis. Firstly, it is the country that has been mostly impacted by the pandemic in terms of total cases and resulting mortality.^4^ Examining how the pandemic has affected the church (UMC) activity levels and finances in the most hit country would certainly be interesting. Secondly, the USA makes a good case for this analysis because it encompasses a blend of cultures and a variety of religions. As a predominately Christian nation, it is home to several dozen Christian denominations. While religions, as a whole, are not homogenous, they do share some similarities in various aspects. All religions and sub-denominations require places of worship, congregants, and active financial support, often through donations from their respective congregations. Therefore, assessing the impact of COVID-19 on a specific religion or religious denomination may have some bearing on other denominations and faiths within the country.

The United Methodist Communications (UMCom) collected and provided the data on the UMC for this analysis.^5^ As an organization, UMCom is responsible for communication, public relations, general funds, and other global programs of the denomination.^6^ UMCom conducted three online surveys (wave 1, wave 2, and wave 3) regarding local church’s responses to the COVID-19 pandemic. The target population included all the United Methodist churches whose emails were registered in an internal UMCom database. The UMCom staff sent survey invitations from their database to the same randomly chosen 11,000 churches in three different periods. Data on church demographic characteristics are presented in Table 1.

**Table 1:** Participant demographic characteristic

	Wave 1	Wave 2	Wave 3	Total
Timeline	26th–30th March	21st–26th April	10th–15th June	
*Church attendance*				
Under 50	327	332	304	
50 to 99	274	276	314	
100 to 249	224	238	270	
250 to 499	82	75	88	
500 or more	54	51	51	
Total	961	972	1027	
*Church location*				
Urban	107	102	157	
Rural	302	294	294	
Small town	338	335	576	
Suburban	213	241	0	
Total	961	972	1027	
Observations	961	972	1027	2963
*Responses*				
Attendance (n)	N/A	964	1005	1969
Finances (n)	952	961	1023	2936
Donations (n)	930	957	1022	2909

The data contain information collected on United Methodist Churches in the USA in three waves. Respondents included church laity and leadership. While we do not have information on the respondents’ age, gender, and race, we do have church location and size information. We also have segregated information on the total responses for our parameters of interest (attendance, finance, and donation) across three waves, as shown in the table below

^a^As the question on church attendance is not a part of wave 1, the regression results are based on waves 2 and 3. This is the reason for relatively lower number of observations

As is evident from Table 1, the first wave of the survey was conducted in March, the second in April, and the third in June 2020. The survey response rate ranged from 8.7% (wave 1) to 9.3% (wave 3), which is not very high. The low return rate might be due to the influx of emails respondents received since the onset of the pandemic (email fatigue). The number of respondents ranged from 961 in wave 1 to 1,030 in wave 3. Since the survey asked the pastors and laity of multi-churches to respond for the larger church they served, there is a possibility that smaller churches might be slightly underrepresented in all three waves. The average weekly church attendance across all three waves is under 50 for most cases (32.5%). While we have respondents from all areas, the majority come from rural and small towns.

All three waves were handled through the *Survey Monkey* platform. Respondents included samples of pastors and congregation leaders.^7^ There is a possibility of a repeated response from the same churches across three waves; however, we do not have this information in the acquired data set. The main variables used and operationalized in this analysis are given below in Table 2.

**Table 2:** Variables and their operationalization

Variable	Type of variable	Measurement	Categories
Time (Wave)	Categorical	Nominal/Ordinal	Wave 1 (26th–30thMarch); Wave 2 (21st–26th April); Wave 3 (10th–15th June)
Location	Categorical Dummy	Nominal Nominal	Urban; Suburban; Rural; Small Town Urban; Non-urban (Suburban + Rural + Small Town)
Attendance	Categorical	Nominal/Ordinal	Lower; Same; Higher; Do not know
Finance	Categorical	Nominal	Finances not a concern; Considering significant changes e–g- layoffs, pay cuts; Tight, but managing by cutting expenses; Might have to close
Donation	Categorical	Ordinal	About the Same; Down; Up

Overall, all three waves included questions aimed at assessing the impact of the pandemic on local church activity levels, finances, and donations. The surveys also sought to determine how local churches were adapting and identify their interest in resources and tools to support such adaptation. The parameters of church adaptation and interest in resources yielded different response options across the three waves. Therefore, for the purpose of this study, we only focus on three parameters of interest: church activity levels (measured by church attendance), church finances, and church donations. By consolidating the respondents from the three waves based on these variables of interest, we obtained a sample of 2,963 churches. The first wave contributed the smallest proportion (32.43%), while the last wave contributed the largest proportion (34.76%) to the consolidated sample. Further details of the data are reported in Table 3.

**Table 3:** Data details

	Wave 1	Wave 2	Wave 3	Total
Timeline	26th–30th March	21st–26th April	10th–15th June	
Observations	961	972	1030	2963
Questions				
Attendance (n)^a^	N/A	964	1005	1969
Finances (n)^b^	952	961	1023	2936
Donations (n)^c^	930	957	1022	2909

*n* Number of observations

^a^The questions used for measuring church attendance in wave 2 and wave 3 are reported below: Wave 3: Is the combined in-person and online average worship attendance for your church higher, about the same, or lower than before the crisis? (a) About the same, (b) Much higher, (c) Much lower, (d). Somewhat higher, (e) Somewhat lower; Wave 2: Is the combined in-person and online average worship attendance for your church higher, about the same, or lower than before the crisis? (a) Higher, (b) Lower, (c) Don’t know, (d). The same for wave 3 answers, we have combined option b and d as “higher” and combined option c and e as “lower”

^b^The question used for measuring financial conditions: How would you describe your church's current financial position? (a) Finances are not a concern, (b) Considering significant changes e–g- layoffs, pay cuts, (c) Tight but we can manage by reducing expenses, (d) Might have to close

^c^The survey questions for donations: Is the giving to your church during the crisis up or down? (a) Up, (b) Down, (c) About the same

## Methods

We employ survey statistical analysis methods in our study. For example, we conduct a Z-tests (Leventhal & Huynh, 1996) to examine the differences in church attendance, church finance, and church donations across time and location. Likewise, we conduct one-way analysis of variance (ANOVA) to determine whether there are any statistically significant differences in church attendance (Heiberger & Neuwirth, 2009; Ross & Willson, 2017), donation, and finances across various locations. Additionally, we employ post hoc Tukey test (Ruxton & Beauchamp, 2008) to identify specific locations that differ significantly from each other in terms of these parameters. Finally, we supplement our analysis with multinomial logistic regressions to ascertain the precise time and location effects (Böhning, 1992). In this analysis, the survey wave and location are treated as independent variables, while church attendance, finance, and donation serve as dependent variables. All of these analyses are performed using STATA 16. Prior to conducting the statistical analyses, we cleaned the data and generated descriptive statistics.

## Statistical Analysis and Results

This section reports the various statistical analyses conducted to investigate the influence of the pandemic on church attendance, church donations, and finances.

### Church Attendance (In-Person + Online)

We begin by examining the changes in church attendance over time. Since the question regarding comparative church attendance in pre-and post-pandemic scenarios was not included in the first wave, we use the data collected in waves 2 and 3. Out of the cumulative 2,002 respondents from waves 2 and 3, a total of 1,969 provided answers to this question. The results are reported in Table 4.

**Table 4:** Church attendance over time

Attendance	Wave 2 (April)	Wave 3 (June)	z-stat (p-value)
Lower	169 (17.53%)	264 (26.27%)	− 4.68*** (0.00)
Same	151 (15.66%)	239 (23.78%)	− 4.52*** (0.00)
Higher	458 (47.51%)	502 (49.95%)	− 1.08 (0.28)
Don’t Know	186 (19.29%)	0	14.63*** (0.00)
Total	964	1005	

z-stat is from a two-tailed test of proportions. *** Significant at 1%

To estimate exclusive time effects, it is necessary to have data from the same churches collected at different time intervals (or balanced panel data) is required. Since we do not have information on whether the same churches responded in waves 2 and 3, comparing church attendance across waves identifies the time effects only partially (with a possibility of bias in the results). However, despite this limitation and the relatively narrow gap of approximately two months between waves 2 and 3, the data still allow us to partially examine how church attendance responds to the pandemic over time.

Interestingly, the proportion of churches reporting a decrease in attendance significantly increased over time (17.5% in wave 2 versus 26.3% in wave 3; p <  = 0.01) even though nearly 30% of the churches reported a shift to online services. This may be partly attributed to the reopening up of the economy, the normalization of business activities, and a higher number of people teleworking, which leaves less time for worship. On the other hand, lower attendance may also be driven, in part, by the fatigue caused by online worship and work during the pandemic.

Next, we explore regional heterogeneity in church attendance by pooling data from the three waves.^8^ We perform a one-way ANOVA test, using church attendance (lower, same, higher, don’t know) as the dependent variable and location (urban, rural, small town, suburban) as the independent variable. The test reveals that church attendance varies significantly across locations (F-stat = 4.38, probability > F = 0.00).

Following the ANOVA, we conduct a post hoc Tukey test to identify which locations differ in terms of church attendance. The test suggests that the difference in church attendance is insignificant between urban and suburban areas.^9^ However, there is a more pronounced and significant difference between urban–rural churches and urban-small town churches.^10^ Conversely, the differences between rural, small-town, and suburban areas are not significant. (The complete details of the post hoc Tukey’s test are reported in Appendix B.)

Since there are no significant differences between rural, small-town, and suburban locations, we combine them into one category, ‘non-urban,’ and further analyze variations in different aspects of church attendance between urban and non-urban areas, employing Z-tests. While the proportion of respondents reporting the same or lower church attendance does not significantly differ across the urban and non-urban areas, the proportion of respondents reporting higher church attendance is higher in urban areas compared to non-urban areas (56.59% versus 47.57%; *p* value = 0.007). Complete test results are reported in Table 5.

**Table 5:** Church attendance across locations

Attendance	Urban	Non-Urban	z-stat (p-value)
Lower	54 (20.93%)	379 (22.15%)	0.44 (0.66)
Same	44 (17.05%)	346 (20.22%)	1.19 (0.23)
Higher	146 (56.59%)	814 (47.57%)	− 2.7***(0.007)
Don’t Know	14 (5.4%)	172 (10.05%)	2.37** (0.02)
Total	258	1711	

Z-stat is from a two-tailed test of proportions. *** Significant at 1%; ** Significant at 5%

Due to the pandemic, people generally stayed home and used online services offered by the church. This effect is expected to be particularly prominent in urban areas because internet penetration and usage are higher in those areas. Also, people in urban areas may have fewer alternative means of socializing amid the pandemic. These factors may have contributed to higher church attendance in urban areas.

To further analyze the results, we conducted a multinomial logistic regression analysis, examining each category within the non-urban specification separately. The results are presented in Table 6. The dependent variable is church attendance, with the base category being “same as prior to pandemic.” The independent variables are location with the base category being urban areas, and wave, which is a binary variable equal to 1 for wave 3 and 0 for wave 2.

**Table 6:** Multinomial logistic regression results for the church attendance

Church Attendance	Higher	Lower	Same	Don’t Know
Base = Urban			Base	
Rural	− 0.468**	− 0.263		0.301
	(0.204)	(0.241)		(0.359)
Small town	− 0.413**	− 0.100		0.228
	(0.193)	(0.226)		(0.354)
Suburban	0.212	0.419		0.333
	(0.287)	(0.334)		(0.416)
Wave 3	− 0.238*	0.102		− 18.42***
	(0.133)	(0.157)		(0.141)
Constant	1.352***	0.136		− 0.0450
	(0.195)	(0.231)		(0.329)
Observations	1,968	1,968	1,968	1,968
Pseudo R-sq	0.067			

Robust standard errors in parentheses

^***^p < 0.01, ** p < 0.05, * p < 0.1

The regression result confirms the earlier findings that church attendance is higher in urban areas compared to rural areas. Ceteris paribus, the log-odds of higher versus same church attendance decrease by 0.47 when moving from urban to rural areas. Similarly, attendance is higher in urban compared to small towns. Ceteris paribus, the log-odds of higher versus same church attendance decrease by 0.41 if we move from urban to a small town. On the other hand, there are no significant differences between urban and suburban areas. Also, in wave 3, a lower proportion of respondents indicate higher attendance compared to wave 2. Holding other factors constant, the log-odds of higher versus same church attendance are 0.24 times lower in wave 3 compared to wave 2. Finally, there are no significant location or time differences between churches reporting lower attendance compared to those reporting the same attendance.

### Donations

Next, we examine the effects of time and location on donations to the church. Although the majority of people responded to this question, 54 respondents did not answer the question regarding the status of donations to the church. Across all three waves, most churches (54.5%) reported a decrease in donations during the pandemic. However, over time, there was an increase in donations, as the proportion of churches reporting an increase in donations rose significantly from 1.08% in wave 1 to 14.09% in wave 3 (results reported in Table 7).

**Table 7:** Donations to churches over time

Giving to church	Wave 1 (March)	Wave 2 (April)	Wave 3 (June)	z-stat (p-value) Wave 1 vs 3
About the same	211 (22.69%)	409 (42.74%)	492 (48.14%)	− 11.70*** (0.00)
Down	709 (76.24%)	490 (51.20%)	386 (37.77%)	17.10*** (0.00)
Up	10 (1.08%)	58 (6.06%)	144 (14.09%)	− 10.65*** (0.00)
Total	930	957	1022	

Z-stat is from a two-tailed test of proportions from the comparison of wave 1 and wave 3. *** Significant at 1%

We report a comparison of donations between wave 1 and wave 3 in Table 7 because these waves are significantly farther apart from each other. The statistical comparison between wave 1 and wave 2 also yields similar results (reported in Appendix C). It is important to acknowledge that we do not have balanced panel data, which means that the results for time effects may be biased.

To examine regional differences in church donations, we conduct a one-way ANOVA test. In this test, donations (about the same, down, up) serve as the dependent variable and location (urban, rural, small town, suburban) is the independent variable. The test indicates insignificant differences (F-stat = 1.20, probability > F = 0.308).

To further analyze the differences between urban and other areas, the regions are categorized into a binary variable consisting of urban and non-urban areas. The non-urban classification includes small cities, small towns, and suburban and rural areas. (Detailed breakdown of donations in each non-urban area is reported in Appendix D.) Interestingly, the comparison between urban and non-urban regions reveals that the proportion of churches reporting an increase in donations is higher in the urban areas (10.25%) compared to non-urban areas (6.87%). Complete results are reported in Table 8.

**Table 8:** Donations to churches across locations

Giving to church	Urban (%)	Non-Urban (%)	z-stat (p-value)
About the same	37.40	38.34	0.35 (0.73)
Down	52.35	54.79	0.87 (0.38)
Up	10.25	6.87	− 2.31** (0.02)

Z-stat is from a two-tailed test of proportions. *** Significant at 1%; ** Significant at 5%

The increased donations in urban areas may be attributed to increased church attendance compared to non-urban regions (as discussed in Table 6). Also, as churches transition to the online donation system, urban areas are more likely to adopt these systems faster than non-urban areas. As a robustness analysis, we also analyzed the decomposed effect of the non-urban variable on donations using multinomial logistic regressions. The output is reported below in Table 9.

**Table 9:** Multinomial logistic regression results for church donations

Donations	Same	Down	Up
Base = Rural	Base		
Urban		− 0.0548	0.581**
		(0.138)	(0.247)
Small town		− 0.173*	0.287
		(0.0980)	(0.193)
Suburban		− 0.107	0.709**
		(0.132)	(0.304)
Base = Wave 1			
Wave 2		− 1.030***	1.086***
		(0.103)	(0.353)
Wave 3		− 1.442***	1.921***
		(0.108)	(0.348)
Constant		1.303***	− 3.413***
		(0.102)	(0.365)
Pseudo R-sq	0.072		
Observations	2,906	2,906	2,906

Robust standard errors in parentheses

^***^ p < 0.01, ** p < 0.05, * p < 0.1

The regression results further support the previous findings. Compared to rural areas, both urban and suburban areas have a positive and significant impact on the increase in church donations. Ceteris paribus, the log-odds of higher versus same church donations increase by 0.58 when moving from rural to urban areas and increase by 0.71 when moving from rural to suburban areas. However, the differences between rural and small-town categories are not significant. Similarly, compared to wave 1, wave 2 (coefficient = 1.086) and wave 3 (coefficient = 1.921) have a positive and significant effect on the increase in church donations, indicating that donations are increasing over time.

As a robustness test, we also conducted a multinomial logistic regression, controlling for the effects of church attendance on church donations. These regression results further reinforce the aforementioned findings. Compared to rural areas, both urban and suburban regions exhibit a positive effect on church donations. Additionally, donations increase over time (please see Appendix E for complete results).

### The Effect of the Pandemic on the Financial Condition

Out of the 2,963 respondents, 27 did not provide an answer to the question regarding the church’s financial situation. Table 10 presents an analysis of the financial state of churches over time.

**Table 10:** Financial condition of churches over time

Financial	Wave 1 (March)	Wave 2 (April)	Wave 3 (June)	z-stat (p-value) wave 1 vs. 3
Finances not a concern	143 (15.02%)	293 (30.49%)	367 (35.87%)	− 10.58 *** (0.00)
Considering significant changes, e.g., layoffs, pay cuts	229 (24.05%)	82 (8.53%)	72 (7.04%)	10.51 *** (0.00)
Tight, but managing by cutting expenses	562 (59.03%)	580 (60.35%)	576 (56.30%)	1.23 (0.22)
Might have to close	18 (1.89%)	6 (0.62%)	8 (0.78%)	2.16 ** (0.04)
Total	952	961	1023	

Z-stat is from a two-tailed test of proportions from the comparison of wave 1 and wave 3. *** Significant at 1%; ** Significant at 5%

Overall, the results presented in Table 10 indicate that the church’s financial problems are being mitigated with time. For example, in the first wave, over 15%, while in the third wave, a significantly higher percentage of 36% indicated that finances were not a concern (p < 0.01). Similarly, in the first wave, 24% of the churches considered drastic changes such as layoffs or pay cuts, but this ratio significantly decreased to just 7% in the third wave (p < 0.01).

Furthermore, in the first wave, almost 2% of church pastors mentioned the possibility of church closure. However, this figure decreased significantly to 0.78% in the third wave (p < 0.05).

In column V of Table 10, we report the statistical comparison between wave 1 and wave 3, as there is a relatively substantial gap of 70 days between these two waves. The statistical differences suggest an improvement in financial conditions. A comparison between wave 1 and wave 2 follows a similar pattern (for details, please refer to “Appendix F”). A multinomial logistic regression also supports most of these results (results in Table 11). Here again, we acknowledge that we do not have balanced panel data; therefore, results for time effects may be slightly biased.

**Table 11:** Multinomial logistic regressions for church finances

	Finance not a concern	Significant changes	Tight but managing	Might close
Base = Wave 2				
Wave 3		0.195	0.120	1.166
		(0.228)	(0.123)	(0.946)
Base = Rural Area				
Urban		1.485***	0.536***	− 14.56***
		(0.315)	(0.178)	(0.482)
Small Town		0.818***	0.406***	− 0.389
		(0.255)	(0.125)	(0.659)
Suburban		1.671***	0.793***	0.344
		(0.350)	(0.205)	(1.205)
Base = Higher Church Attendance				
Lower		0.813***	0.307**	2.362**
		(0.238)	(0.148)	(1.123)
Same		− 0.314	− 0.371***	0.636
		(0.294)	(0.134)	(1.420)
Don’t Know		0.485	0.0913	2.997**
		(0.353)	(0.212)	(1.191)
Base = Donations Same				
Down		2.278***	1.217***	16.53***
		(0.229)	(0.121)	(0.361)
Up		− 2.567**	− 1.121***	-0.629***
		(1.020)	(0.174)	(0.230)
Constant		-3.545***	-0.134	-21.04***
		(0.320)	(0.140)	(1.201)
Observations^a^	1,938	1,938	1,938	1,938
Pseudo R-sq	0.115			

Robust standard errors in parentheses

***p < 0.01, **p < 0.05, *p < 0.1

Next, we examine regional heterogeneity in the financial situation of churches. A one-way ANOVA test with the financial condition as the dependent variable and location (urban, rural, small town, suburban) as the independent variable indicates significant differences in church finances across locations (F-stat = 3.91, probability > F = 0.01). However, further probing through a post hoc Tukey’s test reveals that these differences in financial conditions are only significant between rural and suburban areas.^11^

As the regional heterogeneity in church finances is minimal, with no significant differences between the urban and the other regions, we do not further analyze the differences between urban and non-urban areas as reported in previous sections. However, we analyze the regional differences and the effects of church attendance and donations on the financial condition of churches using a multinomial logistic regression (results in Table 11). As church attendance for wave 1 is unavailable, the regression analysis is restricted to wave 2 and wave 3.

The financial condition of the churches does not significantly differ between wave 2 and wave 3. However, there are three notable findings in Table 11. Firstly, holding other factors constant, the log-odds of churches reporting tight finances versus finances not being a concern increase by 0.54 when moving from rural to urban, by 0.41 when moving from rural to small town, and by 0.79 if when moving from rural to suburban area. Similarly, the log-odds of churches reporting significant financial changes are significantly higher in all non-rural areas. These results indicate that churches in urban, suburban, and small-town localities have tighter finances compared to rural areas. Conversely, holding other factors constant, the log-odds of churches reporting potential closure versus finances not being a concern decrease by 14.56 when moving from rural to urban areas. This result suggests that although churches in urban areas face financial conditions, they are less likely to close compared to churches in rural areas.

Secondly, churches with lower attendance (compared to those with higher attendance) tend to have weaker finances and may consider significant changes or even closure. For example, holding other factors constant, the log-odds of churches reporting potential closure versus finances not being a concern increase by 2.36 when moving from churches with higher attendance to those with lower attendance.

Thirdly, churches that receive increased donations are in better financial shape compared to those with lower donations. Holding other factors constant, the log-odds of churches reporting potential closure versus finances not being a concern increase by 16.53 when moving from churches reporting the same level of donations to those reporting lower donations.

These results indicate that churches with higher attendance and more donations are less likely to face closure and severe financial crises. In the discussion section, we further elaborate on the possible reasons behind these findings and their implications.

Given the potential correlation between church attendance, donations, and time effects captured by the waves, multicollinearity is a possible concern in the multinomial logistic regression results. To assess multicollinearity, we used the *collen* command in STATA to identify variables with a variance inflation factor (VIF) > 10. However, none of the four explanatory variables (wave, location, attendance, donations) had a VIF higher than 1.06. This VIF is significantly lower than the generally accepted cutoff of 10 (Takarinda et al., 2016). Hence, multicollinearity is not a concern in the regression results reported above.

## Discussion and Implications

In this study, we analyzed the effects of COVID-19 on UMC attendance, donations, and finances using survey data obtained from United Methodist Communications (UMCom). Our findings indicate that, compared to the pre-pandemic scenario, church attendance experienced a decline over time as the COVID-19 pandemic persisted, particularly in non-urban areas such as suburban, small-town, and rural locations. This decline in church attendance is an intriguing result as it contradicts previous studies that have reported an increase in religious practice during times of calamity (Ai et al., 2005; DeFranza et al., 2020; Osheim, 2008; Poulin et al., 2009; Torabi & Seo, 2004).

Results in our paper differ from the previous studies, likely due to the nature of COVID-19, which necessitates church closure, social distancing, and mask-wearing. As the COVID-19 persisted in the USA, these preventive measures were observed with a greater force (Siedner et al., 2020), resulting in a significant decrease in in-person church attendance. Also, the ongoing rise in COVID-19 cases may have compelled the public to adhere to precautions and refrain from attending religious services in-person. Another contributing factor to the decline in church attendance could be the resumption of economic activities, which may have reduced the time available for worship. These factors appear to be relevant causes of the decreased church attendance during the pandemic.

Regarding regional heterogeneity, the considerable decrease in church attendance observed in non-urban areas seems to be driven by disparities in online church attendance rate. In our data set, 44.5% of urban churches and 36.9% of rural churches report a shift to online worship—a different practice from their pre-pandemic worship setting. These differences in online church attendance are, in turn, closely tied to the existing digital gap prevalent in urban and non-urban areas in the USA.^12^ Apart from the digital disparities, urban churches might have better outreach and disaster management plans compared to non-urban churches, which could attract more congregants to participate online during the pandemic.

Secondly, we have observed a decrease in church donations during the pandemic compared to the pre-pandemic scenario, which presented financial challenges for churches. However, over time, church donations have increased, and financial issues have been mitigated. One possible reason for this rebound is the financial support provided by the government. For example, a significant proportion of churches (43.11%)^13^ in wave 2 reported applying for financial assistance through the CARE Act. Likewise, 43.56% of churches in wave 3^14^ reported applying for and receiving the financial assistance through the CARE Act.^15^

In addition to the US government’s financial assistance, churches have adapted by accepting donations through online payment platforms. Many churches had not used this strategy before the pandemic. For example, 20.37% of churches in wave 2 reported implementing online donation platforms through services like PayPal and other similar platforms since the pandemic.^16^

Both the government support and the adoption of online donation platforms have played a crucial role in mitigating the pandemic’s impact on donations and alleviating financial constraints for churches. These results also highlight that churches, like most other economic organizations, adapt to the changing circumstances. They do so by taking full advantage of the available digital technologies.

Lastly, active finances and donations are essential for all religious faiths and denominations. Congregants, who are the lifeblood of any denomination, contribute most of the finances through active participation in events. The pandemic has significantly affected the financial security of the average person, and the world economy has experienced a massive slump. This crisis has delivered a financial blow to the church and other religious organizations. Understanding the effects of COVID-19 on church financial conditions and donations will guide the analysis of similar outcomes for other religious denominations and faiths. The results presented in this article suggest a short-term effect of the pandemic on church activity levels and finances. However, it will take more time (likely a couple of years) to determine the long-term overall impact with certainty.

From a policy perspective, the results of this paper highlight the importance of using digital platforms for churches to sustain worship and donations, thereby mitigating the adverse effects of the pandemic on their finances. To achieve this, churches need a robust and well-spread digital infrastructure. Furthermore, universal availability and usage of the internet will play a significant role in the practice of religion in the USA and other parts of the world.

The findings in this paper also indicate that the digital divide in the rural and urban areas can be a crucial factor contributing to regional disparities in the practice of religion and the financial situation of the religious organizations. Therefore, both the state and private sectors should work together to ensure that internet availability and access are facilitated to minimize regional disparities in religious practices. The availability of a reliable digital infrastructure is even more vital when religion can serve as a valuable source of solace and resilience for many people across the world.

Next, we discuss the implications of these results for other religions that are similar in nature, both in the USA and globally. While religions differ in their specific beliefs and practices, they also share many common aspects. For example, followers of major Abrahamic religions such as Christianity, Islam, and Judaism require a place of worship, which, in addition to prayer, is a venue for mediation, consolation, interaction, and social cohesion. With churches, mosques, and synagogues transitioning to online platforms during the pandemic, most believers’ houses have turned into sacred places (Bryson et al., 2020). Similarly, followers of various religious faiths experience many similar feelings and challenges during this pandemic. While they suffer from general anxiety due to the prevailing pandemic and experience mental dissociation because of the recurring restrictions and online fatigue due to a surge in online activities, they are in search of solace. In essence, most religions and their followers are in the same situation. Therefore, the results of this paper, which explore attendance and activity levels in the UMC, may have broader applicability to other religions and denominations.

Furthermore, it has been found that religion moderates the effect of infection (Koenig, 2020; Kowalczyk et al., 2020; Modell & Kardia, 2020). However, it is unclear whether religious institutions in the online world (in other words, ‘online religion’) can provide their virtual followers with all the benefits of in-person religious practice, including moderation and consolation, during the COVID-19 pandemic. It is also unknown whether online religious practices can sustain and maintain levels of congregation attendance and participation. Thus, the results of this paper, which investigates attendance, finance, and activity levels in the UMC, offer insights into exploring these critical dimensions of online religion. Future research would analyze other denominations in the USA and worldwide to validate and expand upon these findings.

## Limitations and Suggestions for Further Research

This study has several limitations which should be considered. Firstly, the survey data in this study primarily relied on responses from pastors and laity of multi-churches who responded for the larger church they served, potentially leading to the underrepresentation of smaller churches. Future research could aim to include responses from a more diverse range of church sizes to obtain a more comprehensive understanding of the impact of the pandemic.

Another limitation relates to the potential bias in time effects. It is unclear whether the same churches participated in all survey waves, which could introduce biases in estimating time effects. Researchers should interpret the time effects with caution, considering this limitation.

Furthermore, the paper could have benefited from additional data on church demographics, such as the age of respondents, population dynamics, and characteristics of the communities they serve. Including such information would provide valuable insights into the contextual factors influencing church attendance, donations, and finances.

Additionally, it is important to note that the results may not be generalizable to all religions. Despite similarities, each religion operates in its own framework. Even within Christianity, there are belief systems and practices as diverse as Mormonism, Pentecostalism, Catholicism, and Protestantism. Within Protestantism, the UMC is a particular variety of mainline Protestantism—one which currently has an older population than most Christian groups in the USA today^17^ and is demographically far whiter.^18^ Future research could explore how the UMC’s religious life is shaped by demographic and cultural realities, including factors such as age and race.

Moreover, the UMC faces a crisis as its leadership is deciding how to deal with a rupture on divisions over the LGBTQ issue.^19^ Therefore, alongside demographic factors, political beliefs about LGBTQ rights and other issues may also provide insights into who possesses the technological skills to attend church remotely, who may perceive a higher risk in gathering in groups, and who may be influenced by diverse media sources making contrasting claims about the pandemic. Examining these dynamics can contribute to a deeper understanding of how these factors intersect with church attendance and engagement during challenging times.

Lastly, to enhance the overall understanding of the impact of the pandemic on Christians, future research could incorporate data from other Christian groups, such as the Roman Catholic and Mormon denominations. Drawing comparisons among different Christian denominations would enable more robust claims about the effects of the pandemic on church attendance and finances within the broader Christian context.

## Conclusions

In conclusion, this study on the United Methodist Church in the USA revealed several key findings. Firstly, church attendance decreased over time during the COVID-19 pandemic, with a more significant decline observed in non-urban areas (suburban, small towns, and rural) compared to urban areas. Secondly, church donations and finances experienced a decline during the pandemic, but these challenges improved over time. Notably, Methodist churches in urban areas showed a faster recovery compared to non-urban churches. Lastly, the study found a positive relationship between church attendance, donations, and overall church finances. These findings shed light on the impact of the pandemic on the United Methodist Church and provide insights into the interplay between attendance, donations, and financial stability.

## Data Availability

The data used in this study were acquired from the United Methodist Communications (UMCom) through a formal data acquisition process. Without the permission of UMCom, we cannot share data. Please get in touch with Charles C. Niedringhaus, Sr. Director of Marketing Research and Evaluation UMCom, for data inquiries.

## Appendix A

See Table

**Table 12:** Church attendance based on location

Attendance	Urban	Rural	Small town	Suburban
Lower	54 (20.93%)	117 (20.38%)	215 (23.97%)	47 (19.66%)
Same	44 (17.05%)	125 (21.78%)	193 (21.52%)	27 (11.30%)
Higher	146 (56.59%)	265 (46.17%)	420 (46.82%)	129 (53.98%)
Don’t Know	14 (5.4%)	67 (11.67%)	69 (7.69%)	36 (15.06%)
Total	258	574	897	239

^12^.

## Appendix B

See Table

**Table 13:** Church attendance and location: Tukey’s post hoc estimates

Location	Contrast	Std. Err	t	P > t
Rural vs Urban	0.28***	0.08	3.6	0.002
Small Town vs Urban	0.19**	0.07	2.59	0.047
Suburban vs Urban	0.16	0.09	1.75	0.296
Small Town vs Rural	− 0.09	0.05	− 1.62	0.366
Suburban vs Rural	− 0.12	0.08	− 1.46	0.462
Suburban vs Small Town	− 0.03	0.07	− 0.35	0.985

The Tukey’s post hoc estimates are estimated after one-way ANOVA. *** Significant at 1%; ** Significant at 5%

13.

## Appendix C

See Table

**Table 14:** Time effects on donations to churches: statistical results

Giving to church	Wave 1 (March) (%)	Wave 2 (April) (%)	z-stat (p-value)
About the same	22.69	42.74	− 9.27***(0.00)
Down	76.24	51.20	11.30***(0.00)
Up	1.08	6.06	− 5.81***(0.00)

Z-stat is from a two-tailed test of proportions. *** Significant at 1%

14.

## Appendix D

See Table

**Table 15:** Regional heterogeneity in donations (observations)

Giving to church	Urban	Rural	Small Town	Suburban
About the same	135	321	508	147
Down	189	507	614	273
Up	37	45	107	23
Total	361	873	1229	443

^15^.

## Appendix E

See Table

**Table 16:** Multi-logistic regression for church donation

Donations	Same	Down	Up
Base = Rural			
Urban		− 0.179	0.525**
		(0.165)	(0.258)
Small Town		− 0.233**	0.316
		(0.116)	(0.203)
Suburban		− 0.322*	0.644*
		(0.175)	(0.333)
Base = Wave 2			
Wave 3		− 0.479***	0.792***
		(0.112)	(0.208)
Base = Same Attendance			
Higher		0.256*	0.410**
		(0.134)	(0.199)
Lower		1.121***	− 0.158
		(0.154)	(0.276)
Don’t Know		0.573***	− 0.501
		(0.200)	(0.513)
Constant		− 0.0711	− 2.494***
		(0.145)	(0.289)
Observations	1,948	1,948	1,948
Pseudo R-sq	0.045		

Robust standard errors in parentheses

^***^ p < 0.01, ** p < 0.05, * p < 0.1

^16^.

## Appendix F

See Table

**Table 17:** Pandemic and financial condition of churches: wave 1 vs. wave 2

Financial condition	Wave 1 (March)	Wave 2 (April)	z-stat (p-value)
Finances are not a concern	15.02%	30.49%	− 8.06*** (0.00)
Considering significant changes, e.g., layoffs, pay cuts	24.05%	8.53%	9.20*** (0.00)
Tight, but managing by cutting expenses	59.03%	60.35%	− 0.59 (0.56)
Might have to close	1.89%	0.62%	2.48** (0.01)

Z-stat is from a two-tailed test of proportions. *** Significant at 1% ** Significant at 5%

17.

## Footnotes

1 PEW Research Center. https://www.pewresearch.org/fact-tank/2016/08/24/why-americas-nones-left-religion-behind/.

2 Washington Times. https://www.washingtontimes.com/blog/watercooler/2012/dec/23/84-percent-world-population-has-faith-third-are-ch/.

3 Reuters https://www.reuters.com/article/us-health-coronavirus-usa-church-finance/empty-pews-empty-collection-baskets-coronavirus-hits-u-s-church-finances-idUSKCN21T0EH.

4 The country witnessed a sharp increase in pandemic cases and suffered life loss and an economic downturn. According to *Worldometers*, the USA reported over 107.38 million cases and 1.16 million deaths as of July 15, 2023. https://www.worldometers.info/coronavirus/countries-where-coronavirus-has-spread/.

5 We acknowledge that the data were provided by the UMCom. We are very thankful to Charles C. Niedringhaus, Sr. Director Marketing Research and Evaluation at UMCom, for tremendous support in providing the data used for analysis in this paper.

6 UMCom https://www.resourceumc.org/en/agencies/communications.

7 The surveys do not elicit response from individual worshippers. This does not pose serious issues for the analysis because our focus is not on individual worship, we rather examine in-person and online church attendance.

8 The details about church attendance based on the locality are reported in “Appendix A”.

9 A post hoc Tukey’s test indicates that the differences between urban and suburban areas are not significant at 10% (t-stat = 1.75, p-value = 0.30).

10 The post hoc Tukey’s test shows that the differences between urban and rural are significant at 1% (t-stat = 3.60, p-value = 0.00) while those between urban and small town are significant at 5% (t-stat = 2.59, p-value = 0.05).

11 A post hoc Tukey’s test indicates that the differences between rural and suburban areas are significant at 1% (t-stat = 3.31, p-value = 0.00).

12 According to the PEWS Research Center, the digital gap is more pronounced in urban and rural areas in the USA, with rural households being 12% less likely to have broadband connections compared to the national average. https://www.pewresearch.org/fact-tank/2019/05/31/digital-gap-between-rural-and-nonrural-america-persists/.

13 In wave 2, 419 churches from a total of 972 report they applied for the assistance through CARES Act. However, we do not have information whether application for assistance was approved or not.

14 A total of 1,001 respondents answered the question about the funding provided by the CARES Act. Out of 1,001 respondents, 436 reported acquiring support through the CARES Act.

15 Wave 1 did not elicit information about the CARES Act because the data collection for wave 1 started before the act was finalized. For further details about the CARE Act, please visit the official government website at: https://home.treasury.gov/policy-issues/cares.

16 This question is not in wave 3.

17 According to the PEW Research Center, only about one-in-ten adults affiliated with the UMC are under the age of 30. The median age of UMC adults is 57. https://www.pewresearch.org/fact-tank/2016/07/11/which-u-s-religious-groups-are-oldest-and-youngest/.

18 According to the PEW Research Center, the membership of the UMC is predominantly white, with more than 90% identifying as white. https://www.pewresearch.org/fact-tank/2015/07/27/the-most-and-least-racially-diverse-u-s-religious-groups/#:~:text=Members%20of%20the%20Evangelical%20Lutheran,all%20more%20than%2090%25%20white.

19 Read this article which says that the UMC voted to maintain its conventional take against same-sex marriage: https://www.christianitytoday.com/news/2019/february/united-methodist-lgbt-vote-conference-plan.html.
